# On the measurement of globe temperatures: analysis of the influence of different parameters

**DOI:** 10.1186/2046-7648-4-S1-A14

**Published:** 2015-09-14

**Authors:** A Virgílio M Oliveira, Adélio R Gaspar, António M Raimundo, Divo A Quintela

**Affiliations:** 1 Coimbra Institute of Engineering, Polytechnic Institute of Coimbra, Department of Mechanical Engineering, Coimbra, Portugal; 2 ADAI-LAETA, Department of Mechanical Engineering, University of Coimbra, Coimbra, Portugal

## Introduction

The assessment of thermal stress, either due to heat or cold, requires the measurement of different physical parameters. Budd [1] states that such assessments are difficult, expensive and time-consuming. The accurate evaluation of the physical parameters is recognized as one of the most important reasons for this statement. When heat stress is considered, the Wet Bulb Globe Temperature index (WBGT) [2] is probably the most common index used throughout the world. Its assessment requires the measurement of the globe temperature, an important issue that is addressed in the present paper.

## Methods

To study the performance of globe thermometers three sensors were used: a 50 mm globe made of a 0.3 mm cooper plate; and two 150 mm standard globes, one from Brüel & Kjær [3] made of a 0.4 mm copper plate and another from Testo with an unknown thickness. The experimental setup also includes a helical fan from Sodeca (type 90/50) to create flows with different air velocities and a radiative heat source with 14 lamps from Philips, type 13117, positioned at different distances from the temperature sensor.

## Results

The analysis of the results considers the response time, the temperature difference between globes and the influence of the air velocity. For the 50 mm globe the response times ranged between 9 and 44 minutes while for the 150 mm globes the values varied from 12 to 48 minutes. The temperature differences between globes are sometimes significant. In the case of the difference between the globe from Testo and the 50 mm globe the lowest difference was 7.5 °C while the highest was 21.7 °C. The analysis of the influence of the air velocity shows that the highest temperature difference between the 150 and 50 mm globes always corresponded to a velocity of 0.5 m.s^-1^, then decreasing for higher air velocities.

## Discussion

The literature suggests that the globe sensors response time is usually between 20 and 30 minutes. The present results alert for the fact that that limit can be overcome. If the temperature difference between the 150 and 50 mm globes is considered, the results highlight that the difference can be important. The use of smaller globes is common due to their advantages, namely in the reduction of the response time. However, when small globes are used the globe temperature is lower, thus calling for the need to correct the values for the standard 150 mm globe, an important matter that is not always addressed. The analysis of the influence of the air velocity clearly identifies the need to separate the natural and forced convection results.

## Conclusion

As a first approach, the measurement of the globe temperature seems simple. Nevertheless, its measurement demands certain requisites for the estimation of the WBGT. This paper presents some precautions to be considered.
